# Majorization and Dynamics of Continuous Distributions

**DOI:** 10.3390/e21060590

**Published:** 2019-06-14

**Authors:** Ignacio S. Gomez, Bruno G. da Costa, Maike A. F. dos Santos

**Affiliations:** 1 Instituto de Física, Universidade Federal da Bahia, Rua Barao de Jeremoabo, Salvador–BA 40170-115, Brazil; 2 Instituto Federal de Educação, Ciência e Tecnologia do Sertão Pernambucano, BR 407, km 08, Petrolina 56314-520, Pernambuco, Brazil; 3 Centro Brasileiro de Pesquisas Físicas, Caixa Postal 15051, Rio de Janeiro CEP 91501-970, RJ, Brazil

**Keywords:** continuous majorization, ordered chain, convex functions, *H*-theorem

## Abstract

In this work we show how the concept of majorization in continuous distributions can be employed to characterize mixing, diffusive, and quantum dynamics along with the *H*-Boltzmann theorem. The key point lies in that the definition of majorization allows choosing a wide range of convex functions ϕ for studying a given dynamics. By choosing appropriate convex functions, mixing dynamics, generalized Fokker–Planck equations, and quantum evolutions are characterized as majorized ordered chains along the time evolution, being the stationary states the infimum elements. Moreover, assuming a dynamics satisfying continuous majorization, the *H*-Boltzmann theorem is obtained as a special case for ϕ(x)=xlnx.

## 1. Introduction

The concept of majorization has shown a particular interest in the last decades, mainly due to its wide range of applicability in information and quantum theory, among others [1,2,3,4,5,6,7,8,9]. The majorization is an operation between a pair of finite-dimensional vectors that gives a partial order in a finite dimensional vectorial space. When the finite vectors considered are discrete probability distributions, the majorization adopts the intuitive idea that, given two discrete PDFs, the distribution which is majorized represents the probability vector of more spreading of the pair, and consequently it presents the lowest Shannon entropy. Further developments showed an intimately relation between majorization and Schur-convex functions [10,11,12,13,14,15,16], from which subsequent applications in quantum information protocols showed the majorization between two quantum states to be an important criteria to establish [17]. Discrete majorization has also been employed for characterizing double stochastic matrices and maps [18], Boltzmann complexity [19], uncertainty relations [20], and generalized entropies [21], among others.

Based on the majorization of discrete finite-dimensional vectors, Hardy, Littlewood and Pólya introduced the continuous version for integrable functions [22,23], giving as result a characterization of the convex ordering for random variables in the context of order statistics. In general, applications of stochastic orders are established from the viewpoint of the continuous majorization [24,25]. In this sense, both types of majorization, the discrete and the continuous ones, provide different ways to relate the increasing direction of the majorization ordering with the monotonic behavior (increasing or decreasing) of convex functionals defined over probability distributions.

The goal of this paper is to study the relationship between the increasing direction of the majorization ordering and the temporal evolution of a continuous dynamics in order to characterize mixing dynamics, diffusion phenomena and quantum evolutions. Thus, the present contribution also could shed light towards a geometrical definition of the arrow time in the context of continuous majorization.

The work is structured as follows. In Section 2, we review the concept of continuous majorization of integrable functions, along with some properties. Then, in Section 3, we consider a general motion equation for a continuous probability distribution and we establish necessary and sufficient conditions for the set of time-parameterized distributions of a given initial solution, that results to be ordered chain by majorization. We characterize the stationary and the initial states as the infimum and the supreme ones of all ordered chain by continuous majorization, respectively. Section 4 is devoted to illustrate the scope of the formalism presented. First, we consider a continuous dynamical system and we obtain a necessary condition for mixing in terms of majorization. Second, we characterize generalized Fokker–Planck equations (without drift terms) as totally ordered chains (by the time evolution) of probability distributions, and their associated Fisher information are also obtained. Third, quantum evolutions (unitary and non-unitary) are explored from the viewpoint of continuous majorization, and a characterization of the *H*-theorem in terms of majorization is given. Next, we illustrate some consequences of continuous majorization applied to the Frobenius–Perron operator associated to a model of exponential population dynamics. Finally, in Section 5, some conclusions and perspectives are outlined.

## 2. Majorization of Integrable Functions and Discrete Vectors

Here, we give the necessary elements for the development of the present work. We begin by recalling the concept of continuous majorization along with some properties.

### 2.1. Continuous Majorization

We say that a real function f(x) is convex (concave, respectively) on a real interval *I* if for all x,y∈I we have f(αx+βy)≤αf(x)+βf(y) (≥, respectively). Consider the set $L^{1}\left(\left(0,1\right)\right)$ of all real Lebesgue integrable function on (0,1) and denote by $L_{\mathrm{cx}}\left(I\right)$ the set of all convex functions on *I*. Given $f,g:I\to R\in L^{1}\left(\left(0,1\right)\right)$, it is said that *f* is *majorized* by *g*, denoted by f≺g, if [22,23,26]

(1) $$\int_{0}^{1}\phi \left(f\left(t\right)\right)dt\leq \int_{0}^{1}\phi \left(g\left(t\right)\right)dt\;,\;\forall \;\phi \in L_{\mathrm{cx}}\left(I\right)$$

whenever the integrals exist. When $L_{\mathrm{cx}}\left(I\right)$ is substituted by $L_{\mathrm{icx}}\left(I\right)$ (denoting the increasing convex functions on *I*), the partial order obtained is *weak majorization*, symbolized by ${≺}_{W}$. It can be seen that ≺ is a reflexive and a transitive relation (i.e., for all $f,g,h\in L^{1}\left(0,1\right)$ we have f≺f and if f≺g,g≺h⇒f≺h) in $L^{1}\left(0,1\right)$. Moreover, ≺ is a partial order since f≺g and g≺f do not imply necessarily f=g a.e. If a set of distributions D has two elements g,h such that g≺f≺h for all f∈D, then it is said that *g* and *h* are the *infimum* and the *supreme* of D, respectively. We say that D is an *ordered chain* by majorization if for all f,g∈D we have f≺g or g≺f.

### 2.2. Discrete Majorization

Complementary, Hardy also defined the discrete majorization and showed its relation with the continuous case, as follows. Let $x,y\in R^{n}$ be two *n*-dimensional vectors. Then, we say that x≺y iff

(2) $$\begin{array}{c} S_{k:n}\left(x\right)\leq S_{k:n}\left(y\right),\;1\leq k\leq n,\;\mathrm{with}\;S_{n:n}\left(x\right)=S_{n:n}\left(y\right)\\ \mathrm{where}\;S_{k:n}\left(z\right)=\sum_{i=n+1-k}^{n}z_{i:n},\;z\in R^{n} \end{array}$$

Here, $z_{1:n}\leq z_{2:n}\leq \ldots \leq z_{n:n}$ denote the components of z arranged in increasing order. The discrete majorization is a partial order in the set $\left\{z\in R^{n}:z_{i}\leq z_{i+1}\;|\;i=1,\ldots ,n-1\right\}$ since from x≺y and y≺x it follows that y is a permutation of x.

Two important concepts that are related to the convexity are the Schur-convexity and the quasi-convexity. A function $f:R^{n}\to R$ is said to be *Schur-convex* if for all $x,y\in R^{n}$ such that x is majorized by y we have f(x)≤f(y). A function $f:R^{n}\to R$ is *quasi-convex* if for all a∈R the preimage of the set (−∞,a) is convex. It can be shown that every convex and symmetric function is Schur-convex but the reciprocal is not true, although all Schur-convex functions are symmetric. The relationship between the continuous majorization and the discrete one is described by the following result [22,23,24,25].

**Theorem** **1.**  *Let $x,y\in R^{n}$ be two n-dimensional vectors and I⊆R an interval. Then, the following propositions are equivalent (see Remark 1.1. of [26]):*

*(a)*  *x is majorized by y.*
*(b)*  *ϕ(x)≤ϕ(y) for each Schur-convex function $\phi :I^{n}\to R$.*
*(c)*  *ϕ(x)≤ϕ(y) for each symmetric quasi-convex function $\phi :I^{n}\to R$.*
*(d)*  *$\sum_{i=1}^{n}\phi \left(x_{i}\right)\leq \sum_{i=1}^{n}\phi \left(y_{i}\right)$ for each convex function g:I→R.*

Note that (d) is the discrete version of the continuous majorization in Equation (1). If necessary, this is the equivalence that we use.

### 2.3. Probability Distributions and Majorization

When the functions or vectors represent probability distributions, the majorization adopts an intuitive interpretation. To illustrate this, we consider the discrete version and the same argument can be applied to the continuous case.

Let $p=(p_{1},\ldots ,p_{n})$ and $q=(q_{1},\ldots ,q_{n})$ be two *n*-dimensional probability distributions, i.e., $p_{i},q_{i}\geq 0$ for all i=1,…,n and $\sum_{i=1}^{n}p_{i}=\sum_{i=1}^{n}q_{i}=1$. It is clear that $p_{i},q_{i}\leq 1$ for all i=1,…,n and there exist $p_{k},q_{l}/p_{k},q_{l}\geq \frac{1}{n}$, thus, by the definition of majorization in Equation (2), we have

(3) $$\left(\frac{1}{n},\ldots ,\frac{1}{n}\right)≺p,q≺\left(1,\ldots ,0\right),$$

where $\left(\frac{1}{n},\ldots ,\frac{1}{n}\right)$ and (1,…,0) are the uniform and delta distributions. This means that, considering ≺ as a partial order on the space of *n*-dimensional probability vectors, the uniform and the delta distributions are the infimum and the supreme elements, respectively.

Physically, if we have an experiment with *n* possible outcomes $x_{i}$ represented by the space of events $\Gamma =\{x_{1},\ldots ,x_{n}\}$ and p is a probability vector (with $p_{i}$ the probability of that *i*th outcome occurs) then discrete majorization says that the distribution with the minimal information (the uniform one) is majorized by p, and in turn, p is majorized by the maximal information distribution (the delta one). Here, the expression “maximal (or minimal) information” is understood in the sense of of maximal (minimal) measure of information (typically, the entropy) available by the system in terms of the probability distribution. In the next sections, we exploit this idea for characterizing temporal evolutions of continuous distributions from majorization.

## 3. Temporal Evolution of Continuous Distributions from Majorization

In this section, we consider a system described by a continuous distribution p(x,t) containing its maximal information about the dynamics at time *t* where *x* represents a continuous variable in (0,1). We focus our study on the relationship between the dynamics and majorization (expressed by the Definition (1)) restricted to the set of time-parameterized distributions $P=\{p_{t}:t\geq 0\}$ with $p_{t}$ the probability distribution of the system at time *t*, i.e., $p_{t}=p\left(x,t\right)$. Given an arbitrary dynamics, the set P provides the evolution of the system from t=0 to t=∞. A first consequence from continuous majorization applied to $P=\{p_{t}:t\geq 0\}$ is given by the following Lemma.

**Lemma** **2.**  *The following statements are equivalent:*

*(I)*  *$P=\{p_{t}:t\geq 0\}$ is an ordered chain by majorization with $p_{t_{2}}≺p_{t_{1}}$ for all $t_{1}\leq t_{2}$ (i.e., the distribution at time t is majorized by all the precedent ones).*
*(II)*  *The function $\lambda_{\phi }\left(t\right):\left[0,\infty \right]\to R$, $\lambda_{\phi }\left(t\right)=\int_{0}^{1}\phi \left(p_{t}\left(x\right)\right)dx$ is decreasing in t for all $\phi \in L_{\mathrm{cx}}\left(I\right)$.*

*In turn, (I) or (II) implies that $\lambda_{\phi }^{\prime }\left(t\right)=\int_{0}^{1}\phi^{\prime }\left(p_{t}\left(x\right)\right)\left(\partial p_{t}/\partial t\right)dx\leq 0$ for all t and for all differentiable $\phi \in L_{\mathrm{cx}}\left(I\right)$, and that the initial distribution $p_{0}$ is the supreme of P.*

**Proof.**  (I)⟹(II): Let $t_{1},t_{2}\geq 0$ be such that $t_{1}\leq t_{2}$. Due to the hypothesis (I), we have that $p_{t_{2}}≺p_{t_{1}}$, which, by the continuous majorization definition in Equation (1), implies that $\int_{0}^{1}\phi \left(p_{t_{2}}\left(s\right)\right)ds\leq \int_{0}^{1}\phi \left(p_{t_{1}}\left(s\right)\right)ds$ for all convex function $\phi \in L_{cx}\left(I\right)$, i.e., $\lambda_{\phi }\left(t_{2}\right)\leq \lambda_{\phi }\left(t_{1}\right)$. Hence, $\lambda_{\phi }\left(t\right)$ is decreasing in *t*.

(II)⟹(I): Reciprocally, if $\lambda_{\phi }\left(t\right)$ is decreasing in *t*, then, for all $t_{1}\leq t_{2}$, we have $\lambda_{\phi }\left(t_{2}\right)\leq \lambda_{\phi }\left(t_{1}\right)$, which by definition means that $\int_{0}^{1}\phi \left(p_{t_{2}}\left(s\right)\right)ds\leq \int_{0}^{1}\phi \left(p_{t_{1}}\left(s\right)\right)ds$ for all convex function $\phi \in L_{cx}\left(I\right)$. Then, $p_{t_{2}}≺p_{t_{1}}$ and thus the set $P=\{p_{t}:t\geq 0\}$ is an ordered chain by majorization.

Finally, let us assume (II) and consider $\phi \in L_{cx}\left(I\right)$ differentiable. Since $\lambda_{\phi }\left(t\right)=\int_{0}^{1}\phi \left(p_{t}\left(x\right)\right)dx$ is a decreasing function in *t*, its derivative must be negative for all *t*, thus $\lambda_{\phi }^{\prime }\left(t\right)=\int_{0}^{1}\phi^{\prime }\left(p_{t}\left(x\right)\right)\left(\partial p_{t}/\partial t\right)dx\leq 0$. By the equivalence between (I) and (II) we have that $p_{t}$ is majorized by all their precedent ones. In particular, it follows that $p_{t}≺p_{0}$ for all t≥0 so $p_{0}$ is the supreme of P. This completes the proof. □

The content of Lemma 2 is that, when we have a dynamics satisfying (I), it can be characterized by the increasing behavior of the functions $\lambda_{\phi }\left(t\right)=\int_{0}^{1}\phi \left(p_{t}\left(x\right)\right)dx$ for all convex function ϕ, where the initial distribution majorizes all the subsequent evolved ones. Thus, a first simple connection between dynamics and continuous majorization is provided. We show that the hypothesis (I) is compatible with the intuitive idea that, in diffusion phenomena, as the distribution evolves, it tends to spread along its domain (thus approaching to the uniform one that is the infimum element). This is precisely the content of the next result.

**Lemma** **3.**  *Assuming hypothesis (I) of Lemma 2 and the existence of an asymptotic probability distribution at t→∞, denoted by $p_{\infty }$ satisfying $\partial p_{\infty }/\partial t=0$, we have*

(4) $$p_{\infty }≺p_{t}≺p_{0}\;,\;\forall \;t\geq 0$$

*and*

(5) $$\lambda_{\phi }^{\prime }\left(t\to \infty \right)=0\;,\;\forall \;\phi \in L_{\mathrm{cx}}\;\mathrm{differentiable}$$

**Proof.**  Due to (I), we have $p_{t_{2}}≺p_{t_{1}}$ for all $t_{1}\leq t_{2}$. By definition, $p_{\infty }=\lim_{t\to \infty }p_{t}$, from which we obtain $p_{\infty }≺p_{t}$ for all t≥0. From condition (I) of Lemma 2, it follows that $p_{0}$ is the supreme of P. Joining these conditions, we obtain $p_{\infty }≺p_{t}≺p_{0}$ for all t≥0. Now, given that the asymptotic probability distribution $p_{\infty }$ satisfies $\partial p_{\infty }/\partial t=0$, by Lemma 2, for all differentiable $\phi \in L_{cx}$, we have $\lambda_{\phi }^{\prime }\left(t\to \infty \right)=\lim_{t\to \infty }\int_{0}^{1}\phi^{\prime }\left(p_{t}\left(x\right)\right)\left(\partial p_{t}/\partial t\right)dx=\int_{0}^{1}\phi^{\prime }\left(p_{\infty }\left(x\right)\right)\left(\partial p_{\infty }/\partial t\right)dx=\int_{0}^{1}\phi^{\prime }\left(p_{\infty }\left(x\right)\right).0dx=0$. In turn, this implies that $\lambda_{\phi }\left(t\right)$ takes an asymptotic finite value for t→∞ for all differentiable $\phi \in L_{cx}$, as physically expected for the stationary probability distribution $p_{\infty }$. □

When the time evolution preserves majorization in the sense of condition (I), the Lemmas 2 and 3 allow to characterize the dynamics in terms of continuous majorization, where the initial state is the supreme and the stationary one is the infimum. In particular, for the Shannon–Gibbs entropy functional S[p]=−∫ψ(p(x))dx given by the convex function ψ(x)=xlnx, condition (II) says that $-\lambda_{\psi }\left(t\right)$ is an increasing function of *t*, thus $-\lambda_{\psi }\left(t_{1}\right)\leq -\lambda_{\psi }\left(t_{2}\right)$$\forall t_{1}\leq t_{2}$, i.e., $S\left[p_{t_{1}}\right]\leq S\left[p_{t_{2}}\right]$$\forall t_{1}\leq t_{2}$, in accordance with the Second Law of thermodynamics.

Next step is to study what kind of phenomena can be compatible with continuous majorization.

## 4. Applications

To study what type of dynamics can be obtained from continuous majorization, in this section, we illustrate the results with some examples. We begin by discussing the H-Boltzmann theorem. In all the examples, the abstract space *X* of the variable *x* is the real interval (0,1), in order to be compatible with the definition of the continuous majorization in Equation (1).

### 4.1. *H*-Theorem and Majorization

We explore the relationship between continuous majorization and one of the pillars of the statistical mechanics: the *H*-theorem. In 1872, Ludwig Boltzmann introduced a functional *H* to describe, in an elegant way [27], the approach to equilibrium of a gas composed by colliding molecules in a finite volume with perfectly elastic walls. Let us assume that the gas is sufficiently dilute and that only binary collisions are needed to describe the dynamics. Let p(r,v,t) be the distribution function of the molecules such that $p\left(r,v,t\right)d^{3}rd^{3}v$ is the number of molecules within a volume $d^{3}r$ centered at r with a velocity in a volume $d^{3}v$ centered at v. We can also consider that p(r,v,t)=p(r,t)p(v) which physically means the correlations between the positions and the velocities are negligible when the gas is diluted (here, p(r,t) and p(v) are the marginal distributions with respect the positions and the velocities). Under the SZA (“Stosszahlansatz") hypothesis about the number of collisions for all times, we have that the H-functional

(6) $$\begin{array}{c} H\left[p_{t}\right]=\int p\left(r,v,t\right)\ln p\left(r,v,t\right)d^{3}rd^{3}v=\int p\left(r,t\right)\ln p\left(r,t\right)d^{3}r+\mathrm{constant} \end{array}$$

satisfies

(7) $$\begin{array}{c} \frac{dH}{dt}\leq 0, \end{array}$$

where $p_{t}=p\left(r,t\right)$ and the integration in Equation (6) is over all the positions. The statement of Equation (7) constitutes the *Boltzmann’s H-theorem* [28] (or simply *H*-theorem). *H*-theorem provides a justification to the equilibrium approach and the increasing of the associated entropy *H* (called “negentropy”, negative of the thermodynamical entropy), and more fundamental, negentropy can be defined by means of Equation (6) for distributions out of the equilibrium. Let us show how the *H*-theorem can be obtained as a special case of a dynamics satisfying continuous majorization. This is the content of the following result.

**Theorem** **4.**  *If dynamics is preserved by majorization in the sense of condition (I) (or equivalently, condition (II) of Lemma 2), then the H-theorem is satisfied.*

**Proof.**  To compatibilize with Equation (1), we consider that the position space $X=\{r:r\in R^{3}\}$ is the unidimensional interval (0,1) (the set I=(0,1) of the continuous majorization definition in Equation (1) can be replaced by any subset bounded interval (a,b)⊆R and all the definitions and results remain valid, as can be checked straightforwardly). This situation fits with the image of a diluted gas with all its particles contained in a unidimensional box. If condition (I) is satisfied for the set $P=\{p_{t}:t\geq 0\}$ with $p_{t}=p\left(x,t\right)$, then, by Lemma 2, for $\phi_{0}\left(s\right)=s\ln s$ (which is a convex function), we have that $\lambda_{\phi_{0}}^{\prime }\left(t\right)\leq 0$ with $\lambda_{\phi_{0}}\left(t\right)=H\left(t\right)$. This implies that $\frac{dH}{dt}\leq 0$ for all t≥0. □

### 4.2. Dynamical Systems: Mixing Property

One of the central concepts of dynamical systems theory and statistical mechanics is the mixing condition [29,30], i.e., the asymptotic vanishing of the correlations between two subsets of phase space that are sufficiently separated in time. In the usual definition in the language of distribution functions, this reads as

(8) $$\begin{array}{c} \exists f_{*}\in L^{1}\left(X\right)\;\mathrm{such}\mathrm{that}\;\forall f\in L^{1}\left(X\right),g\in L^{\infty }\left(X\right):\\ \lim_{t\to \infty }\int_{X}f\left(T_{t}\left(x\right)\right)g\left(x\right)dx=\int_{X}f_{*}\left(x\right)g\left(x\right)dx \end{array}$$

where *X* is the phase space, $f_{*}$ is the equilibrium distribution of the system at t→∞ ($f_{*}\circ T_{t}=f_{*}$), and $T_{t}:X\to X$ is a continuous transformation, typically the Liouville time evolution in classical mechanics. In particular, Equation (8) says that the measure $\mu_{*}\left(A\right)=\int_{A}f_{*}\left(x\right)dx$ is invariant under $T_{t}$.

Now, let us assume the dynamics satisfies condition (I) of Lemma 2 and X=(0,1). In particular, for the convex functional ϕ(x)=|x|, we have $||f\circ T_{t}{\left|\right|}_{1}\geq ||f\circ T_{t^{\prime }}{\left|\right|}_{1}$ for all $t\leq t^{\prime }$ where ${\left||\phi |\right|}_{1}=\int_{X}\left|\phi \left(x\right)\right|dx$ is the 1-norm. It follows that $\int_{X}|\left(f\circ T_{n}\right)\left(x\right)g\left(x\right)|dx\leq ||f\circ T_{n}{\left|\right|}_{1}{\left||g|\right|}_{\infty }$ $\leq ||f\circ T_{0}{\left|\right|}_{1}{\left||g|\right|}_{\infty }={\left||f|\right|}_{1}{\left||g|\right|}_{\infty }$ for all n∈N, where we have used the *Hölder–Minkowski inequality* [31] and $f\circ T_{0}=f$. Thus, the sequence $\int_{X}\left(f\circ T_{n}\right)\left(x\right)g\left(x\right)dx$ is bounded and then there exists $\lim_{n\to \infty }\int_{X}\left(f\circ T_{n}\right)\left(x\right)g\left(x\right)dx$ for all $g\in L^{\infty }$. Thus, by application of the *Riesz representation theorem* [31] to the functional $\psi \left(g\right):L^{\infty }\mapsto R$ defined by $\psi \left(g\right)=\lim_{n\to \infty }\int_{X}\left(f\circ T_{n}\right)\left(x\right)g\left(x\right)dx$, we have that there exists $f_{*}\in L^{\infty }$ such that $\psi \left(g\right)=\langle f_{*},g\rangle =\int_{X}f_{*}\left(x\right)g\left(x\right)dx$ for all $g\in L^{\infty }$. Hence, the system is mixing. We can see that continuous majorization guarantees that the asymptotic distribution $f_{*}$ belongs to $L^{\infty }$, which means that this is bounded almost everywhere in the phase space.

### 4.3. Generalized Fokker–Planck Equations

Disordered and thermal molecular motion is macroscopically characterized as diffusion phenomena of a net flux of particles from one region to other. Under Markovian assumptions and making the passing to the continuum, the discretized master equation for the probability transition states becomes the Fokker–Planck equation (FPE) for the probability distribution. Recently, a generalization of the FPE [32,33,34] (recovering the nonlinear and linear cases as special ones) that links generalized entropic forms with the theorem *H* is proposed in the form [35,36]

(9) $$\frac{\partial p(x,t)}{\partial t}=-\frac{\partial \{F(x)\Psi [p(x,t)]\}}{\partial x}+\frac{\partial }{\partial x}\left\{\Omega \left[p\left(x,t\right)\right]\frac{\partial p(x,t)}{\partial x}\right\}$$

where p(x,t) is the probability distribution of the particle at time *t*, $F\left(x\right)=-\frac{d\varphi }{dx}$ is a conservative force acting over the particles, and Ω[p],Ψ[p]>0 are nonnegative functionals. From the functional $\lambda_{\phi }\left(t\right)$, we can relate majorization with the generalized FPE in Equation (9) as follows. Considering F(x)=0, we have $\int^{x}ds\frac{\partial p(s,t)}{\partial t}=\Omega \left[p\left(x,t\right)\right]\frac{\partial p(x,t)}{\partial x}$ with Ω[p(x,t)]>0. Thus, if we integrate by parts the derivative of the function $\lambda_{\phi }\left(t\right)$ (i.e., $\lambda_{\phi }^{\prime }\left(t\right)$), we have

(10) $$\begin{array}{c} \lambda_{\phi }^{\prime }\left(t\right)={\left[\phi^{\prime }\left(p_{t}\left(x\right)\right)\int^{x}ds\frac{\partial p(s,t)}{\partial t}\right]}_{0}^{1}\\ -\int_{0}^{1}\phi^{\prime \prime }\left(p_{t}\left(x\right)\right)\frac{\partial p(x,t)}{\partial x}\left(\int^{x}ds\frac{\partial p(s,t)}{\partial t}\right)dx \end{array}$$

where the first term can be neglected since $\frac{\partial p(x,t)}{\partial x}=0$ at x=0,1. Replacing this in Equation (10) and using that $\phi^{\prime \prime }\geq 0$, we conclude

(11) $$\begin{array}{c} \lambda_{\phi }^{\prime }\left(t\right)=-\int_{0}^{1}\phi^{\prime \prime }\left(p_{t}\left(x\right)\right)\Omega \left[p\left(x,t\right)\right]{\left(\frac{\partial p(x,t)}{\partial x}\right)}^{2}dx\leq 0 \end{array}$$

for all $\phi \in L_{\mathrm{cx}}\left(I\right)$ differentiable. Hence, the generalized FPE in Equation (9) with F(x)=0 satisfies the condition (I) of Lemma 2. This means that for F(x)=0 the solutions $p_{t}=p\left(x,t\right)$ of the generalized FPE in Equation (9) constitute an ordered chain by majorization.

### 4.4. Quantum Dynamics

We analyze how the continuous majorization can characterize quantum dynamics. We consider that the set P is given by the evolution of the eigenfunctions probability distributions, i.e., $P_{n}=\left\{\right|\psi_{n}\left(x,t\right){|}^{2}:t\geq 0\}$ with *n* the energy index,

(12) $$i\hbar \frac{\partial \psi_{n}}{\partial t}=H\psi_{n}=E_{n}\psi_{n}$$

and

(13) $$E_{n}=\epsilon_{n}+i\gamma_{n}$$

Equation (13) expresses the fact that the Hamiltonian *H* may be non-Hermitian, for instance in open quantum systems [37]. In the non-Hermitian case, the measurable eigenergies of the system are the $\epsilon_{n}$ while the $|\gamma_{n}{|}^{2}$ are proportional to the decay times. It is clear that the usual unitary case is recovered when $\gamma_{n}=0$ for all *n*. To verify if the dynamics prescribed by Equation (12) preserves majorization according to condition (I) of Lemma 2, we calculate the derivate of the function $\lambda_{\psi_{n}}\left(t\right)$ for each energy index *n*. Doing this and using that $\frac{d\psi_{n}}{dt}\psi_{n}^{*}+\frac{d\psi_{n}^{*}}{dt}\psi_{n}=2\frac{\gamma_{n}}{\hbar }{\left|\psi_{n}\right|}^{2}$, we obtain

(14) $$\lambda_{\phi }^{\prime }\left(t\right)=\frac{2\gamma_{n}}{\hbar }\int_{0}^{1}\phi^{\prime }\left(\right|\psi_{n}{|}^{2})|\psi_{n}{|}^{2}dx\;\;,\;\;\forall \;\phi \in L_{\mathrm{cx}}\left(I\right)\;\mathrm{diff}$$

where the domain of the variable *x* of the eigenfunctions $\psi_{n}\left(x,t\right)$ is assumed to be (0,1). Equation (14) is the starting point for characterizing some types of quantum dynamics in terms of majorization.

*Case I: Hermitian dynamics $\gamma_{n}=0\;\forall n$:* From Equation (14), we can see that $\lambda_{\phi }^{\prime }\left(t\right)=0$ for all $\psi_{n}$, thus $\lambda_{\phi }\left(t\right)$ is constant for all *t*, which implies that, for all $\psi_{n}\left(x,t_{1}\right),\psi_{n}\left(x,t_{2}\right)$ and $t_{1},t_{2}$, we have that $|\psi_{n}\left(x,t_{1}\right){|}^{2}≺{\left|\psi_{n}\left(x,t_{2}\right)\right|}^{2}$ and $|\psi_{n}\left(x,t_{2}\right){|}^{2}≺{\left|\psi_{n}\left(x,t_{1}\right)\right|}^{2}$. This means that the infimum and the maximum are always the same $|\psi_{n}{\left(x,t\right)|}^{2}$ (with t≥0 arbitrary) along time. In other words, in a unitary dynamics, the the order relation of the continuous majorization becomes the trivial one.

*Case II: Non-Hermitian dynamics $\gamma_{j}\neq 0$ for some j:* Given $\phi \in L_{\mathrm{cx}}\left(I\right)$ differentiable, since ϕ is convex, $\phi^{\prime \prime }$ is nonnegative, which implies that $\phi^{\prime }$ is increasing. In particular, from $|\psi_{n}{\left(x,t\right)|}^{2}\geq 0$, it follows that $\phi^{\prime }\left(\right|\psi_{n}{|}^{2})\geq \phi^{\prime }\left(0\right)$. Assuming $\gamma_{j}<0$ for some *j*, we obtain

(15) $$\lambda_{\phi }^{\prime }\left(t\right)\leq \frac{2\gamma_{j}}{\hbar }\phi^{\prime }\left(0\right)\;,\;\forall \;\phi \in L_{\mathrm{cx}}\left(I\right)\;\mathrm{differentiable}$$

In this case, we see that, to fulfill condition (I), we need $\phi^{\prime }\left(0\right)\geq 0$ ($\phi^{\prime }\left(0\right)\leq 0$ if $\gamma_{j}>0$, respectively) for all $\phi \in L_{\mathrm{cx}}\left(I\right)$, which can be satisfied if $\phi \in L_{\mathrm{icx}}\left(I\right)$, thus leading to a weak majorization. Hence, for the case of a non-Hermitian dynamics, we have that $P_{j}=\left\{\right|\psi_{j}\left(x,t\right){|}^{2}:t\geq 0\}$ is a ordered chain by weak majorization when $\gamma_{j}<0$.

### 4.5. Population Dynamics: Exponential Model and Majorization

Now, we show an application of continuous majorization to the exponential model of population dynamics. This model is given by the discrete map

(16) $$\begin{array}{c} N_{k+1}=\lambda N_{k}\;,\;\forall k=0,1,\ldots \end{array}$$

where $N_{k}$ is the number of individuals of the population at a discrete time *k*, $N_{0}$ represents the initial population, and the parameter λ≥0 defines the population growth rate. The dynamics is completely characterized by four regimes:

λ=1:  Since $N_{k}=N_{0}$ for all *k*, the population remains the same along time.
λ<1:  The number of individuals decreases in each time step so it tends to zero for large times.
λ>1:  The number of individuals is growing in such a way that it tends to infinity asymptotically.
λ=0:  This case correspond to the extinction of the population since $N_{k}=0$ for all time *k*.

Beyond the simplicity of the exponential model, it is instructive to investigate a characterization from the viewpoint of the continuous majorization. Thus, we are interested in studying the dynamics by means of probability densities instead of trajectories $\left(N_{0},N_{1},\ldots ,N_{k},\ldots \right)$. This is according with the more realistic situation where the exact number of individuals at a given time is unknown and only known to be distributed over a range of values. If $\rho_{0}\left(x\right)$ represents mathematically how the initial population is distributed and *x* is the initial number of individuals, then we can analyze the dynamics in terms of continuous majorization by means of the transfer operator. In the following, we employ some definitions and concepts presented in Chapter 3 of the book by Mackey and Lasota [38].

Given a discrete map $x_{k+1}=f\left(x_{k}\right)$ with f:X→X and *X* the variable space of the map (typically, a subset of the real numbers), the transfer operator *P* associated (Frobenius–Perron operator, equivalently) is defined as (see Equation (3.2.2) of [38])

(17) $$\begin{array}{c} \int_{A}P\rho \left(x\right)dx=\int_{f^{-1}\left(A\right)}\rho \left(x\right)dx \end{array}$$

for all continuous probability distribution ρ(x):X→R and subset A⊆X, being $f^{-1}\left(A\right)$ the preimage of *A*. The Frobenius–Perron operator *P* belongs to the class of Markov operators, which is a relevant family of operators in dynamical systems theory since they allow to define the evolution of distributions functions on abstract spaces.

For unidimensional maps and when *f* is a differentiable and bijective function, given a probability distribution ρ:X→R, the Frobenius–Perron admits the following expression (see Equation (3.2.7) of [38])

(18) $$\begin{array}{c} P\rho \left(x\right)=\rho \left(f^{-1}\left(x\right)\right)\left|\frac{d}{dx}f^{-1}\left(x\right)\right| \end{array}$$

For the case of the exponential model, from Equation (16), we have f(x)=λx and $f^{-1}\left(x\right)=\lambda^{-1}x$. Then, the associated Frobenius–Perron operator takes the form

(19) $$\begin{array}{c} P\rho \left(x\right)=\lambda^{-1}\rho \left(\lambda^{-1}x\right) \end{array}$$

Let us show that the Frobenius operator in Equation (19) generates a dynamics that preserves continuous majorization in Equation (1) for all initial distribution. Let ϕ:R→R be a convex function with ϕ(0)=0. Due to the convexity, for all 0<α≤1, we have

(20) $$\begin{array}{c} \alpha^{-1}\phi \left(\alpha y\right)\leq \phi \left(y\right)\leq \alpha \phi \left(\alpha^{-1}y\right) \end{array}$$

for all y∈R. Since λ=0 and λ=1 correspond to the two trivial stationary cases, it is sufficient to only consider 0<λ<1 and λ>1. Let g(x) be a probability distribution defined on an interval [a,b]. Then, by Equation (19), the evolved probability distribution Pg is defined over [f(a),f(b)]. Here, we also consider the possibility that the interval of integration *I* in the definition of the continuous majorization in Equation (1) can be the set of real numbers, i.e., I=R, which is according to the Definition 2.6. of [39].

We begin by $\underline{0<\lambda <1}$: Taking $y=g(\lambda^{-1}x)$ and α=λ, from Equation (19) and using the right side of the inequalities in Equation (20), it follows that

(21) $$\lambda^{-1}\phi \left(g\left(\lambda^{-1}x\right)\right)\leq \phi \left(\lambda^{-1}g\left(\lambda^{-1}x\right)\right)=\phi \left(Pg\left(x\right)\right);$$

then, by integrating Equation (21) between f(a)=λa and f(b)=λb, we obtain

(22) $$\int_{I}\phi \left(g\left(s\right)\right)ds=\int_{a}^{b}\phi \left(g\left(s\right)\right)ds=\int_{\lambda a}^{\lambda b}\lambda^{-1}\phi \left(g\left(\lambda^{-1}x\right)\right)dx\leq \int_{f(a)}^{f(b)}\phi \left(Pg\left(s\right)\right)ds=\int_{I}\phi \left(Pg\left(s\right)\right)ds,$$

which shows that *g* is majorized (with [a,b],[f(a),f(b)]⊆I=R and g,Pg naturally extended over R as being zero outside their respective domains) by Pg, i.e., g≺Pg. In turn, this can be interpreted as the probability distribution Pg is less spread than *g*. This means that, when the growth of the rate is higher than 1, any initial probability distribution *g* results less widespread along its domain as it evolves, and therefore a localization around some region of the domain takes place.

Case $\underline{\lambda >1}$: Now, we consider $y=g(\lambda^{-1}x)$ and $\alpha =\lambda^{-1}$. Then, from Equation (19) and the left side of the inequalities in Equation (20), we have

(23) $$\phi \left(Pg\left(x\right)\right)=\phi \left(\lambda^{-1}g\left(\lambda^{-1}x\right)\right)\leq \lambda^{-1}\phi \left(g\left(\lambda^{-1}x\right)\right)$$

thus, integrating both sides of Equation (23) between f(a)=λa and f(b)=λb, we obtain the inequality in Equation (22) but in the opposite order, i.e.,

(24) $$\int_{I}\phi \left(Pg\left(s\right)\right)ds=\int_{f(a)}^{f(b)}\phi \left(Pg\left(s\right)\right)ds\leq \int_{a}^{b}\phi \left(g\left(s\right)\right)ds=\int_{I}\phi \left(g\left(s\right)\right)ds.$$

This means that, when λ>1, according to Equation (1), *g* majorizes Pg (Pg≺g) and then any initial probability distribution *g* spreads along its evolution, thus showing a diffusive behavior as the time increases. Thereby, the characterization of the Frobenius–Perron operator in Equation (19) associated to the map in Equation (16) in terms of continuous majorization allows obtaining features of the dynamics without performing any explicit calculus but providing general conditions.

Let us illustrate the localized (0<λ<1) and diffusive (λ>1) behaviors of the probability distributions when the evolution is given by the Frobenius–Perron operator in Equation (19) with a concrete example. We consider that our ignorance about the initial population is given by a uniform distribution $\rho_{0}\left(x\right)=1_{\left[0,1\right]}\left(x\right)$ along the interval [0,1]. Here, the dimensionless variable *x* represents the fraction of individuals with respect to a maximum allowed number of individuals *M*. We denote $P^{n}\rho_{0}\left(x\right)=P\circ \cdots_{n\mathrm{times}}\cdots \circ P\rho_{0}\left(x\right)$ as $\rho_{n}\left(x\right)$, which represents the probability distribution of the population after *n* time steps. Using Equation (19), we obtain

(25) $$\begin{array}{c} \rho_{n}\left(x\right)=P^{n}\rho_{0}\left(x\right)=\frac{1}{\lambda^{n}}1_{\left[0,\lambda^{n}\right]}\left(x\right) \end{array}$$

which expresses different behaviors depending on the value of the growth rate λ. For instance, when λ=1, the initial population distribution remains the same $P^{n}1_{\left[0,1\right]}\left(x\right)=1_{\left[0,1\right]}\left(x\right)$ for all time *n*.

**Figure 1:**
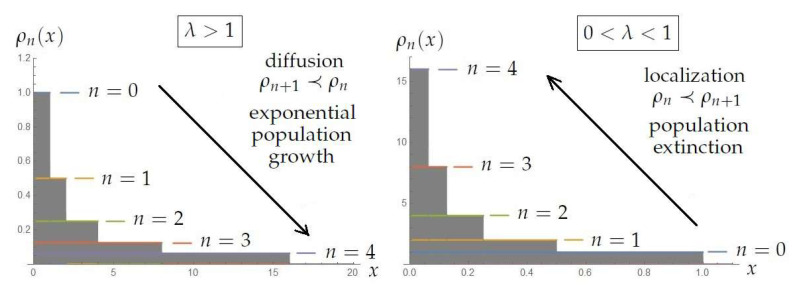
Evolved probability distributions $\rho_{n}\left(x\right)$ given by the Equation (25) for λ=2 and λ=1/2, respectively. The arrow indicates the temporal evolution. The diffusive regime represents an increasing of the ignorance about the population and the majorization ordering in Equation (1) is opposite to the temporal one (arrow). By contrast, when localization occurs, the population rapidly concentrates around x=0, which expresses the extinction of the population, and the majorization ordering coincides with the temporal evolution.

In Figure 1, we illustrate the two relevant regimes (0<λ<1 and λ>1) in terms of the probability distribution $\rho_{n}$ for n=0,1,2,3,4 and for some the representative values of λ=2,1/2. We can see the fast diffusion or localization (around x=0, i.e., population extinction) of the the probability distribution $\rho_{0}$, previously characterized by means of majorization.

## 5. Conclusions

By considering the concept of continuous majorization in ordered chains, whose elements are continuous distributions representing solutions of physical equations, in this paper, we have explored some relationships that illustrate the role played by continuous majorization in different contexts. We have obtained simple results (Lemmas 2 and 3) that allow characterizing the dynamics towards the equilibrium for all system whose states are represented by probability distributions, where the temporal order can be expressed univocally as a majorization order, in which, as the distribution evolves along the time, it is majorized by its corresponding earlier times. As a result of this characterization, the equilibrium distribution and the initial one correspond to the minimum and maximal elements, respectively.

After this characterization of a continuous dynamics by means of continuous majorization, we have analyzed some different examples. Specifically, under the assumption of a dynamics satisfying continuous majorization (condition (I) of Lemma 2), the *H*-theorem has been obtained as a special case by choosing the convex function ϕ(x)=xlnx (Theorem 4). Analogously, the mixing property in dynamical systems has been obtained by assuming a dynamics satisfying condition (I) and choosing ϕ(x)=|x|. For the case of the Curado–Nobre generalized FPE in absence of forces, we have shown that the set of solutions indexed by the time constitutes an ordered chain by continuous majorization. Last, we have explored the quantum dynamics generated by the probability distributions given by the eigenfunctions in terms of continuous majorization. In this case, we have found that the Hermitian (unitary) case corresponds to the trivial order while the non-Hermitian (non-unitary) case gives a non-trivial order.

**Figure 2:**
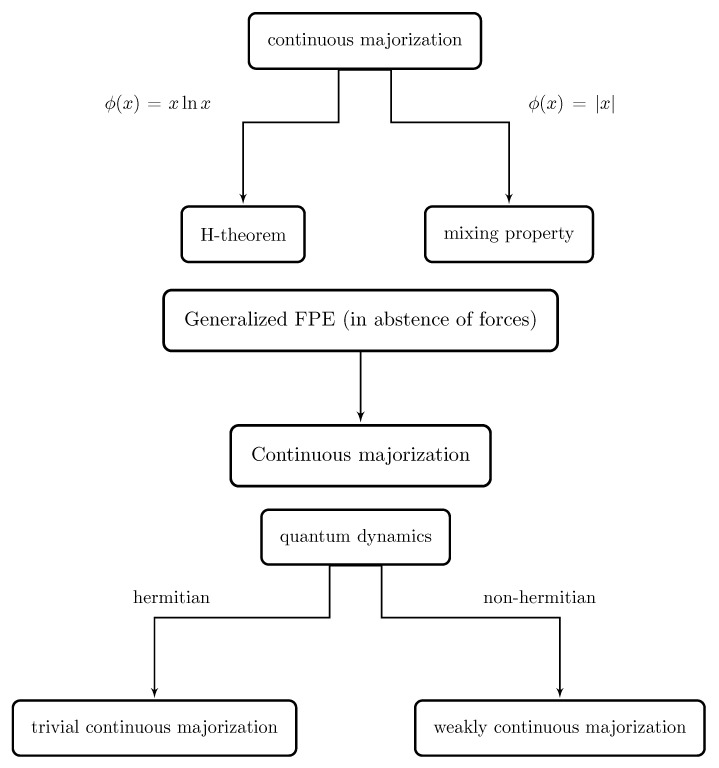
Some necessary and sufficient conditions for continuous majorization in different contexts illustrate the relevance of the concept of majorization in a continuous dynamics.

In summary, our contribution is two-fold. On the one hand, Lemmas 2 and 3 provide a general framework for majorization in a continuous dynamics that can be exported in specific contexts, as illustrated in Section 4. On the other hand, the linking between continuous majorization and the *H*-theorem by means of Theorem 4 could serve to shed light on the question of the thermodynamical arrow of time. In this sense, we can see that the continuous majorization contains a proper arrow of time governed by its induced order in a continuous dynamics. It is worth noting that the continuous majorization removes some relevance of the thermodynamical entropy as the main functional to be considered; for instance, in the context of dynamical systems, we have seen that the convex function to obtain the mixing property corresponds to the module function. This kind of flexibility by means of the choice of the convex function ϕ is schematized in Figure 2. After this, we have also provided an example with a simple model of exponential population dynamics. In this example, the concept of continuous majorization applied to the Frobenius–Perron allowed us to characterize the relevant regimes of the population dynamics by obtaining general conditions (diffusion and localization), which were subsequently illustrated (Figure 1).

Finally, an examination of these consequences with more examples is expected in future contexts (for instance, in fractional nonlinear systems [40], quantifying chaos [41], integro-differential equations [42], etc.) in order to establish with more details the role of continuous majorization on the foundations of the physics.
